# Inequality-Related Health and Social Factors and Their Impact on Well-Being during the COVID-19 Pandemic: Findings from a National Survey in the UK

**DOI:** 10.3390/ijerph18031014

**Published:** 2021-01-24

**Authors:** Daniel Tzu-Hsuan Chen, Yi-Jen Wang

**Affiliations:** 1 Public Health Policy Evaluation Unit, School of Public Health, Imperial College London, London W6 8RP, UK; yi-jen.wang18@imperial.ac.uk; 2 Department of Family Medicine, Taipei Veterans General Hospital, Taipei 112, Taiwan

**Keywords:** COVID-19, health inequalities, social determinants, pandemic, population health, well-being

## Abstract

*Background:* Lower socioeconomic groups and disadvantaged populations across the world suffer disproportionately from the coronavirus disease 2019 (COVID-19) pandemic. This study aimed to examine the impact of health- and social-inequality–related factors on well-being in order to further distinguish each of their effects during the pandemic. *Methods:* A nationally-representative sample of 5077 UK respondents aged 18 years or older was recruited through an online survey panel during the COVID-19 pandemic. Their subjective well-being was measured using the 11-point Cantril Ladder of Life Scale. The impact of inequality-related health and social factors (pre-existing medical conditions, household size and occupation), as well as COVID-19–related risk factors (symptoms, confirmed infections, and social distancing behaviours) on well-being were analysed using multiple linear regression models. The associations between the COVID-19–related risk factors and well-being according to the respondents’ household size and occupation were modelled in order to test the differences by their socioeconomic profile. *Results:* We identified inverted V-shaped associations between household size and subjective well-being during the COVID-19 pandemic. Compared to single-person households, respondents from households of two to four persons showed better well-being (β = 0.57; CI (0.44, 0.72)), whereas living in crowded households of five persons or more was associated with decreased well-being (β = −0.48; CI (−0.71, −0.25)). Furthermore, lower-skilled occupations (elementary occupations: β = −0.31; CI (−0.58, −0.03); logistics and transport services: β = −0.37; CI (−0.74, −0.01)) and chronic medical conditions (cardiometabolic or respiratory diseases: β = −0.25; CI (−0.41, −0.1); and mental health conditions: β = −1.12; CI (−1.28, −0.96)) were factors associated with reduced well-being during the pandemic. Interactions between a positive COVID-19 diagnosis, symptoms, and crowded households were identified (β = −0.95; CI (−1.76, −0.14) and β = −4.74; CI (−9.87, −1.61), respectively). *Conclusions:* In a national sample, the levels of general subjective well-being during the COVID-19 pandemic and lockdowns were disproportionately distributed across different groups within society. Preventive policies should explicitly focus on reaching lower socioeconomic groups; more emphasis should be placed on the coordination of multisectoral support in order to tackle existing health and social inequalities.

## 1. Introduction

The World Health Organization has declared the novel coronavirus disease of 2019 (COVID-19) a global pandemic, as it has spread rapidly with high fatality rates across the world [1]. The European region, including the United Kingdom (UK), has been severely affected by the coronavirus outbreak, with waves of death and confirmed cases. The virus has shown that it respects no borders; however, it does affect people unequally. Consolidated evidence has indicated increased vulnerability to the virus among the most socially and economically disadvantaged, and COVID-19 has disproportionately affected these populations’ health and well-being across the world [2,3,4].

Recent studies on inequalities in COVID-19 have highlighted multiple factors contributing to the differential vulnerability to the infection and the consequences of the disease [5,6]. Inequalities in socioeconomic status (SES)—measured by economic deprivation, poor housing conditions, or employment in precarious occupations—have been linked to increased exposure to respiratory diseases [3,5]. These social inequalities translate into inequalities in health, as lower SES is correlated with poorer general health and a higher prevalence of pre-existing conditions (e.g., cardiovascular diseases, respiratory diseases, diabetes, and cancer) [7], making such low SES groups highly susceptible to COVID-19 infection [3,4]. Altogether, these potential risk factors show that disadvantaged populations suffer a disproportionately heavier burden from the consequences of the pandemic.

However, evidence on how the disease strikes certain populations more adversely, and its impact on well-being at the population level during the pandemic, remain scarce. As many of the potential health and social risk factors are intertwined, it is important to distinguish each of their relative effects in order to inform the preventive policies tackling existing inequalities in the era of COVID-19. This study aimed to examine the impact of inequality-related health and social factors, as well as COVID-19–related risk factors on subjective well-being in the UK during the pandemic.

## 2. Methods

The data were collected via YouGov COVID-19 behaviour tracker data [8], which contain international, anonymised, respondent-level data from an ongoing web-based weekly survey during the COVID-19 pandemic. We analysed the data from the UK, with a pooled sample of 5077 respondents, aged 18 years or older, who were surveyed between 30 April and 31 May 2020, when a national lockdown was implemented in the UK. The panel of participants were matched to the UK census data and then weighted by age, gender, and region in order to construct representative samples of the UK population. All of the data are publicly available and fully anonymised; thus, no ethical approval was required for the present analysis.

The respondents self-reported their sociodemographic factors (e.g., age, gender, household size, occupation), health and behavioural factors (e.g., pre-existing conditions, COVID-19 symptoms/test results, social distancing behaviours), and the Cantril Ladder of Life Scale [9]. Due to the unavailability of the data, information about the respondents’ race/ethnicity was not included in the present study. The responses were sorted and merged into broader categories according to official national/international recommendations [10,11]. Household size was categorised into three distinctive groups: households of one person, households of two to four persons, and households of five persons or more. A cut-off point of five persons or more was used to determine crowded living conditions, as households of five or more contribute to most of the overcrowding of households in England [11]. The respondents reported their occupations if they had to work outside of their home in the next seven days at the time of the survey. We categorised the occupations into six groups according to the occupations’ similarity [10]: working at home, elementary occupations (construction, manufacture, and food retail), health and social care services (healthcare and social care), logistics and transport services (logistics, public transport, and delivery services), safety and social services (policing, prisons, and schools), or others (other unspecified occupations that require working away from home). Pre-existing conditions prior to the pandemic were collected and grouped into mental health conditions, cardiometabolic or respiratory diseases (asthma, chronic obstructive pulmonary disease (COPD), cystic fibrosis, heart diseases, high blood pressure, high cholesterol, or diabetes), other diseases (cancer, AIDS/HIV, epilepsy, multiple sclerosis, or arthritis), and none. COVID-19–related questions on whether the respondents had experienced any newly-developed COVID-19–relevant symptoms, or had tested positive for COVID-19, were asked. Social distancing behaviours were measured by asking whether they had been in contact with people outside their households during the lockdown period. Finally, the 11-point Cantril Ladder of Life Scale, abbreviated as the Cantril Ladder, is a validated instrument for the measurement of general subjective well-being, including psychosocial well-being and life satisfaction [9]. The respondents were asked to rate, on an imaginary ladder with steps from 0 (representing ‘the worst possible life’) to 10 (representing ‘the best possible life’), which step of the ladder represents their present life.

Multiple linear regression models accounting for the sample survey weights were used to assess the impact of the identified health and social determinants on well-being. Furthermore, we included interaction terms in order to model associations between COVID-19 symptoms, diagnosis, and well-being according to the respondents’ household size and occupation, with adjustments for age, gender, pre-existing conditions, and social distancing, in order to test the differences of impact by their SES profile. A one-way analysis of variance (ANOVA) was used to detect differences in the mean Cantril Ladder scores among the determinants. All of the statistical analyses were performed using STATA version 13.0, and the bilateral significance level was set to 0.05.

## 3. Results

Of the 5077 respondents, 2472 (46.7%) were male and 2605 (53.3%) were female. The mean Cantril Ladder scores for the subjective well-being of the whole sample were 6.13 ± 1.99 out of 10, and the scores did not significantly differ between the genders (Supplementary Table S1). Significant differences in the Cantril Ladder scores were found between household size groups, occupation groups, those with certain pre-existing conditions (i.e., cardiometabolic or respiratory diseases, mental health conditions) versus none, and those with COVID-19 symptoms and confirmed infection groups. The respondents with mental health conditions reported the lowest mean Cantril Ladder scores (4.88 ± 2.12).

In the multiple linear regression analysis, age, household size, and lower-skilled occupations were factors that were significantly associated with subjective well-being, as assessed by Cantril Ladder scores (Table 1). In particular, age was positively correlated (β = 0.03; CI [0.02, 0.03]) with the scores, while household size demonstrated mixed associations, i.e., the scores were higher among households of two to four persons (β = 0.57; CI (0.44, 0.72)) but lower in households of five persons or more (β = −0.48; CI (−0.71, −0.25)) compared to single-person households. In terms of occupation, the respondents who reported working in elementary occupations, and in logistics and transport services, exhibited lower Cantril Ladder scores (β = −0.31; CI (−0.58, −0.03) and β = −0.37; CI (−0.74, −0.01), respectively) compared to those who worked at home.

Compared to respondents without any pre-existing conditions, those with cardiometabolic or respiratory diseases, as well as those with mental health conditions, presented significantly lower Cantril Ladder scores (β = −0.25; CI (−0.41, −0.10) and β = −1.12; CI (−1.28, −0.96), respectively). Additionally, our results demonstrated that testing positive for COVID-19 (β = −1.05; CI (−2.22, −0.13)) and experiencing newly-developed COVID-19 symptoms (β = −0.33; CI (−0.54, −0.12)) were significantly correlated with lower Cantril Ladder scores. However, reporting adherence to social distancing guidelines was not associated.

No significant interaction was found between COVID-19–related risk factors and occupation (results not shown). The results of the interactions of COVID-19 diagnosis and COVID-19 symptoms with household size in relation to Cantril Ladder scores are presented in Table 2. Positive COVID-19 diagnosis and having COVID-19 symptoms had significant interactions with crowded households on subjective well-being, with lower beta coefficients (β = −0.95; CI (−1.76, −0.14) and β = −4.74; CI (−9.87, −1.61), respectively).

## 4. Discussion

In a national sample, our preliminary analysis indicated that the national average Cantril Ladder scores were generally lower than the widely-accepted cut-off point of 7 [9] across all of the groups during the COVID-19 pandemic. However, the pandemic has had a greater negative impact on well-being among lower-skilled workers, people living in crowded conditions, and those with pre-existing cardiometabolic, respiratory or mental health conditions during the early stage of the pandemic. Our primary findings are in line with national reports on the disparities in the risk and health outcomes of COVID-19 [12]. Accordingly, the pandemic has potentially increased existing health inequalities and mortality rates among people in deprived areas; in Black, Asian and Minority Ethnic (BAME) groups; in precarious occupations; and in those with comorbidities [12]. In particular, our study found that a positive COVID-19 diagnosis and having COVID-19 symptoms had interaction effects with crowded living conditions that deteriorated well-being. Our findings add to the existing literature by analysing the underlying factors in each health and social determinant, as discussed, in order to better understand their distinct impact on the health outcomes of COVID-19.

Living conditions play an essential role in well-being. Interestingly, we identified an inverted V-shaped association between household size and well-being, with respondents living in households of two to four persons showing higher levels of well-being compared to those living in single or crowded households. This finding indicates that small to medium-sized households had facilitative effects on better well-being during the pandemic. This effect may be explained by the social support received from household members during the pandemic, whereas for people who lived alone, such support was reduced due to the lockdown and stay-at-home orders [13] during the survey period. However, this was not the case for respondents living in relatively-crowded households. Living in crowded households of five persons or more caused lower levels of well-being, and such levels were even lower among respondents with coexisting symptoms or a diagnosis of COVID-19. This negative impact is consistent with prior studies that highlight population density and poor living conditions as being detrimental to physical and mental health [14]. This is particularly relevant for people with low levels of SES, as economically-disadvantaged people are more likely to live in conditions that deteriorate psychological distress [15] and the immune system, leading to increased susceptibility to respiratory tract infections [14].

The impact of COVID-19 on well-being also differed by occupation. Lower-skilled workers were among those hit hardest by the pandemic [12]. Our analysis showed that, compared to work-at-home respondents, workers in elementary occupations (i.e., construction, manufacture, and food retail), and logistics, delivery services and public transport were associated with a significant decrease of 0.31 and 0.37 units in well-being, respectively. According to the data from the UK Official for National Statistics, domestic workers, and leisure and other service occupations are significantly more likely to suffer from excessive exposure to COVID-19 due to physical proximity to others; for example, public transport and bus drivers were reported to have the highest rates of mortality from COVID-19 [12]. As financially-disadvantaged people are often employed in occupations that do not provide opportunities to work from home, many of these workers risk their health and well-being performing precarious jobs during the pandemic. However, such differences were not observed among healthcare, social care, safety, and social service workers. Although it is contradictory to the findings on healthcare workers in China [16], our study embodies a broader spectrum of occupations, highlighting the contrast of well-being among lower-skilled workers compared to relatively-higher–skilled workers with less-insecure employment.

The respondents with pre-existing cardiometabolic and respiratory diseases, and those with mental health conditions, experienced a significant plunge in well-being during the pandemic. This was especially prominent in those with mental health conditions, showing a decrease of 1.12 units on the Cantril Ladder score. As the prevalence of chronic conditions is negatively associated with SES [7], the disadvantaged population represents a group at higher risk of being affected by the pandemic. Our findings are consistent with recent studies on the exacerbation of psychosocial disorders among vulnerable populations, such as marginalised individuals or those with pre-existing mental conditions [16,17]. Other studies have reported similar results on the impact of cardiorespiratory and chronic comorbidities on poorer health outcomes from COVID-19 [18]. However, with regard to other diseases, such as cancer or AIDS, no association with well-being was identified in the current study. This may result from the function of our form of measurement. The Cantril Ladder is a fairly general measurement of overall well-being status [9]. Specific aspects of physical and psychological health might be related to specific health problems that are not reflected in our current measurement, which requires further investigation. 

We found no difference in well-being between those who adhered to social distancing guidelines by avoiding contact with people outside their households and those who did not. Although the lockdown measures apply restrictions on in-person socialization, it can be argued that, if the respondents continued to network socially, either face-to-face or remotely, the social distancing rules did not seem to cause changes in their well-being status immediately within the survey period. However, a negative impact on well-being was noted among the respondents with COVID-19–relevant symptoms or with confirmed COVID-19 infection, which is in line with the existing studies [16]. More strikingly, the significant interactions between positive COVID-19 diagnosis or having COVID-19 symptoms and living in crowded households were found to have more detrimental impacts on well-being. While national guidance advised that people with COVID-19 symptoms or positive test results should self-isolate at home and maintain social distancing from others, people within crowded households seemed to experience elevated distress that may have resulted in deteriorated well-being.

Our findings on age and gender showed similar results to previous large-scale international surveys reporting improved life satisfaction and well-being with advancing age in both genders [19,20]. This may be explained by the increased coping ability for adverse life events at an older age. Although one recent review suggested that older adults were at increased risk for mental health concerns during the COVID-19 pandemic [16], limited evidence is available to determine the mental health risks related to age and its associated factors during the pandemic. 

The current study provides further evidence that disadvantaged populations suffer unequally greater well-being–related burdens from the impact of the pandemic. As in the case of the UK, many of these vulnerable populations include key workers during this unprecedented crisis. Timely, clear and coherent guidance should be given to the public in order to mitigate the negative impacts across the population, and to establish preparedness for future crises [2]. Although the policies in individual countries varied in how they sought to mitigate the circumstances of the vulnerable population during the pandemic, preventive policies and multisectoral support should specifically target those in precarious occupations, those in single and crowded households, and those with pre-existing conditions, in order to tackle the existing inequalities incurred by the pandemic. For instance, this could involve identifying people with confirmed COVID-19 infections and then tailoring supportive treatments according to their household conditions, in order to ease family distress and promote individual wellness. Confirmed cases with suboptimal housing conditions may benefit from receiving telehealth services for psychophysical support, as well as support from local authorities to ensure that they receive sufficient health care both physically and mentally during their quarantine and recovery. Some limitations should be noted. All of the data was collected from online self-reported surveys, which inevitably may be at risk of misclassification and response bias. The standardised categorisation of overcrowding using the bedroom standard by official reports [11] was not available due to the limitations of the dataset. Furthermore, as this report is a preliminary investigation, there were several outstanding social determinants of health that we did not account for due to the limited available data. These include the income, educational level, access to healthcare, and ethnic background of the respondents. The further stratification of the demographics and longitudinal follow-up to better identify inequality-associated factors and the long-term effects of this pandemic on individuals’ well-being are urgently needed.

## 5. Conclusions

Disadvantaged populations face a disproportionately-higher risk of poor health and mental outcomes from COVID-19. Our study adds to the existing literature by providing a better understanding of how inequalities during the COVID-19 pandemic are affecting well-being, and it distinguishes each of their relative effects in order to inform the preventive policies tackling existing health and social inequalities. Policies should explicitly focus on reaching low SES groups, and should provide multisectoral support in order to ensure a rapid and coordinated response to protect the disadvantaged in future crises.

## Figures and Tables

**Table 1:** Multiple linear regression models for Cantril Ladder scores among health and social determinants.

	*n* (%)	β	95% Confidence Interval
Age	-	0.03 ***	0.02–0.03
Gender *(ref. male)*			
Female	2605 (53.3)	0.10	−0.01–0.21
Household size *(ref. single person)*			
2 to 4 persons	3680 (72.5)	0.57 ***	0.44–0.72
5 persons or more	390 (7.7)	−0.48 ***	−0.71–−0.25
Don’t know	101 (1.9)	−0.23	−0.63–0.16
Occupation *(ref. work at home)*			
Elementary occupations ^a^	193 (3.8)	−0.31 *	−0.58–−0.03
Logistics and transport ^b^	103 (2.0)	−0.37 *	−0.74–−0.01
Health and social care ^c^	232 (4.6)	0.03	−0.24–0.29
Safety and social services ^d^	114 (2.3)	0.22	−0.15–0.59
Others ^e^	262 (5.2)	−0.19	−0.44–0.05
Prefer not to tell	2354 (46.4)	−0.50 ***	−0.63–−0.38
Cardiometabolic or respiratory diseases ^f^ *(ref. none)*			
Yes	1727 (34.0)	−0.25 **	−0.41–−0.10
Mental health conditions *(ref. none)*			
Yes	620 (12.2)	−1.12 ***	−1.28–−0.96
Others diseases ^g^ *(ref. none)*			
Yes	888 (17.5)	−0.17	−0.41–0.06
COVID-19 symptoms ^h^ *(ref. none)*			
Yes	336 (6.6)	−0.33 **	−0.54–−0.12
Prefer not to tell	97 (1.9)	−0.09	−0.45–0.34
Confirmed COVID-19 infection *(ref. no)*			
Yes	10 (0.2)	−1.05 *	−2.22–−0.13
Prefer not to tell	68 (1.3)	−0.24	−0.76–0.28
Social distancing ^i^ *(ref. not adhered)*			
Adhered to guidelines	3090 (60.9)	−0.08	−0.21–0.06

Note: Cantril Ladder score (min–max: 0–10). Higher scores indicate better general subjective well-being. The model applied accounted for sample survey weights. The weighted percentages are reported for the frequency distributions. ^a^: Construction, manufacture, and food retail; ^b^: logistics, delivery services, and public transport; ^c^: healthcare and social care; ^d^: policing, prisons, and schools; ^e^: other unspecified occupations that require working away from home; ^f^: asthma, chronic obstructive pulmonary disease (COPD), cystic fibrosis, heart diseases, high blood pressure, high cholesterol or diabetes; ^g^: cancer, AIDS/HIV, epilepsy, multiple sclerosis or arthritis; ^h^: experiencing newly-developed symptoms of any of the following: dry cough, fever, loss of sense of smell or taste, shortness of breath or breathing difficulty; ^i^: measured by asking if respondents have been in close contact with people outside their households during the lockdown period. Positive responses were regarded as not having adhered to the guidelines; * *p* < 0.05, ** *p* < 0.01, *** *p* < 0.001.

**Table 2:** Interactions between COVID-19 symptoms, confirmed COVID-19 diagnosis, and household size.

		β	95% Confidence Interval
COVID-19 symptoms ^a^	Household size		
*(ref. none* × *single person)*			
Yes	2 to 4 persons	0.22	−0.32–0.75
Yes	5 persons or more	−0.95 *	−1.76–−0.14
Yes	Don’t know	−1.03	−2.56–−0.49
Prefer not to tell	2 to 4 persons	0.95	0.32–2.22
Prefer not to tell	5 persons or more	−2.82 ***	−4.44–−1.21
Prefer not to tell	Don’t know	−2.27	−0.65–−3.89
Confirmed COVID-19 infection	Household size		
*(ref. no* × *single person)*			
Yes	2 to 4 persons	−2.452	−6.47–1.57
Yes	5 persons or more	−4.74 **	−9.87–−1.61
Yes	Don’t know	-	-
Prefer not to tell	2 to 4 persons	0.460	−1.35–2.27
Prefer not to tell	5 persons or more	−2.00	−4.22–0.211
Prefer not to tell	Don’t know	−1.21	−3.26–0.83

Note: The model accounted for the sample survey weights and was adjusted for age, gender, occupation, cardiometabolic or respiratory diseases, mental health and other conditions, and social distancing. a: Experiencing newly developed symptoms of any of the following: dry cough, fever, loss of sense of smell or taste, shortness of breath or breathing difficulty. -: empty with no observations; * *p* < 0.05, ** *p* < 0.01, *** *p* < 0.001.

## Data Availability

The datasets generated and analysed during the current study are available from: https://github.com/YouGov-Data/covid-19-tracker.

## Supplementary Materials

The following are available online at https://www.mdpi.com/1660-4601/18/3/1014/s1, Table S1: Weighted frequency distributions, means and standard deviations of Cantril Ladder scores.

## Abbreviations

COVID-19	Coronavirus disease 2019
SES	socioeconomic status
COPD	chronic obstructive pulmonary disease
AIDS/HIV	acquired immune deficiency syndrome/human immunodeficiency virus
ANOVA	one-way analysis of variance
BAME	black, Asian and minority ethnic groups.
