# Supplementary material for: Inequality-Related Health and Social Factors and Their Impact on Well-Being during the COVID-19 Pandemic: Findings from a National Survey in the UK
Source: Int J Environ Res Public Health. 2021 Jan 24;18(3):1014. doi: 10.3390/ijerph18031014 (PMC7908210; doi:10.3390/ijerph18031014)
Supplement: Supplementary file 1 [file ijerph-18-01014-s001.pdf]

International Journal of
Environmental Research and Public Health

MDPI

Table S1. Weighted frequency distributions, means and standard deviations of Cantril Ladder scores.

		n (%)	Cantril Ladder score §	*p*-value §§
Gender	Male	2,472 (46.7)	6.11 ± 2.01	0.53
	Female	2,605 (53.3)	6.15 ± 1.97	
Household size	Single person	906 (17.9)	5.89 ± 1.87	<0.01
	2 to 4 persons	3,680 (72.5)	6.28 ± 1.92	
	5 persons or more	390 (7.7)	5.76 ± 2.12	
	Don't know	101 (1.9)	4.94 ± 7.12	
Occupation	Work at home	1,819 (35.8)	6.27 ± 1.70	<0.01
	Elementary occupations [a]	193 (3.8)	5.98 ± 1.85	
	Logistics and transport [b]	103 (2.0)	5.92 ± 1.77	
	Health and social care [c]	232 (4.6)	6.23 ± 1.92	
	Safety and social services [d]	114 (2.3)	6.44 ± 1.68	
	Others [e]	262 (5.2)	6.18 ± 1.77	
	Prefer not to tell	2,354 (46.4)	6.01 ± 2.23	
Cardiometabolic or respiratory diseases [f]	Yes	1,727 (34.0)	6.12 ± 2.05	<0.05
	None	3,350(66.0)	6.16 ± 1.94	
Mental health conditions	Yes	620 (12.2)	4.88 ± 2.12	<0.01
	None	4,457 (87.8)	6.33 ± 1.89	
Others diseases [g]	Yes	888 (17.5)	6.14 ± 1.94	0.06
	None	4,189 (82.5)	6.15 ± 2.14	
COVID-19 symptoms [h]	Yes	336 (6.6)	5.64 ± 2.14	<0.01
	None	4,644 (91.5)	6.18 ± 1.97	
	Prefer not to tell	97 (1.9)	5.40 ± 2.17	
Confirmed COVID-19 infection	Yes	10 (0.2)	6.14 ± 1.98	<0.01
	No	4,999 (98.5)	7 ± 2.70	
	Preferer not to tell	68 (1.3)	5.34 ± 2.25	
Social distancing [i]	Adhered to guidelines	3,090 (60.9)	6.11 ± 2.01	0.35
	Not adhered	1,987 (39.1)	6.16 ± 1.9	

§ Cantril Ladder score (min–max: 0–10). Higher score indicates better general well-being, expressed as mean ± standard deviation. §§ Kruskal–Wallis mean ranks test (>2 group comparison) or Mann–Whitney U test (2 group comparison); a: construction, manufacture, and food retail; b: logistics, delivery services, and public transport; c: healthcare and social care; d: policing, prisons, and schools; e: other unspecified occupations that require working away from home; f: asthma, chronic obstructive pulmonary disease (COPD), cystic fibrosis, heart diseases, high blood pressure, high cholesterol or diabetes; g: cancer, AIDS/HIV, epilepsy, multiple sclerosis or arthritis; h: experiencing newly developed symptoms of any of the following: dry cough, fever, loss of sense of smell or taste, shortness of breath or breathing difficulty; i: measured by asking if respondents have been in close contact with people outside their households during the lockdown period. Positive responses were regarded as not adhered to guidelines.
